# Overexpression of Insig-2 inhibits atypical antipsychotic-induced adipogenic differentiation and lipid biosynthesis in adipose-derived stem cells

**DOI:** 10.1038/s41598-017-11323-9

**Published:** 2017-09-07

**Authors:** Chien-Chih Chen, Li-Wen Hsu, Kuang-Tzu Huang, Shigeru Goto, Chao-Long Chen, Toshiaki Nakano

**Affiliations:** 1 Department of Psychiatry, Kaohsiung Chang Gung Memorial Hospital and Chang Gung University College of Medicine, Kaohsiung, 833 Taiwan; 2 Liver Transplantation Center and Department of Surgery, Division of Transplant Immunology, Institute for Translational Research in Biomedicine, Kaohsiung Chang Gung Memorial Hospital and Chang Gung University College of Medicine, Kaohsiung, 833 Taiwan; 3 Graduate Institute of Clinical Medical Sciences, Chang Gung University College of Medicine, Kaohsiung, 833 Taiwan

## Abstract

Atypical antipsychotics (AAPs) are considered to possess superior efficacy for treating both the positive and negative symptoms of schizophrenia; however, AAP use often causes excessive weight gain and metabolic abnormalities. Recently, several reports have demonstrated that AAPs activate sterol regulatory element-binding protein (SREBP). SREBP, SREBP cleavage-activating protein (SCAP) and insulin-induced gene (Insig) regulate downstream cholesterol and fatty acid biosynthesis. In this study, we explored the effects of clozapine, olanzapine and risperidone on SREBP signaling and downstream lipid biosynthesis genes in the early events of adipogenic differentiation in adipose-derived stem cells (ASCs). After the induction of adipogenic differentiation for 2 days, all AAPs, notably clozapine treatment for 3 and 7 days, enhanced the expression of SREBP-1 and its downstream lipid biosynthesis genes without dexamethasone and insulin supplementation. Simultaneously, protein level of SREBP-1 was significantly enhanced via inhibition of Insig-2 expression. By contrast, SREBP-1 activation was suppressed when Insig-2 expression was upregulated by transfection with Insig-2 plasmid DNA. In summary, our results indicate that AAP treatment, notably clozapine treatment, induces early-stage lipid biosynthesis in ASCs. Such abnormal lipogenesis can be reversed when Insig-2 expression was increased, suggesting that Insig/SCAP/SREBP signaling may be a therapeutic target for AAP-induced weight gain and metabolic abnormalities.

## Introduction

Schizophrenic patients need to take antipsychotic agents for most of their lives. Atypical antipsychotics (AAPs) are considered to possess superior efficacy for treating both the positive and negative symptoms of schizophrenia compared to typical antipsychotics^1, 2^. However, a high prevalence of metabolic syndrome^3–6^ and increased mortality rate^7, 8^ were observed in schizophrenic patients, and were considered to be adverse effects of AAPs. AAP-induced metabolic syndrome consists of excessive weight gain, type II diabetes mellitus, hyperglycemia, dyslipidemia, insulin resistance and cardiovascular disease^3–7, 9, 10^. Of note, the incidence of cardiovascular mortality in the schizophrenic population is over twice that of the general population^5, 11^, which may also be related to AAP treatment. Consequently, it is important to monitor the metabolic function of patients with schizophrenia, who have a higher risk of metabolic disorders due to the use of AAPs.

The mechanisms underlying AAP-induced metabolic dysfunction are still elusive. Several hypotheses have been proposed, including the impact of genetic factors^12, 13^, stimulation of appetite by central histamine H1 blockage^14^, modulation of serotoninergic/noradrenergic pathways in the central nervous system and/or involvement of the hormones leptin, ghrelin and adiponectin^15^. Additionally, AAPs are involved not only in the regulation of critical adipose biochemical processes, including signal transduction, mitochondrial biogenesis, adipogenesis and metabolism during adipogenic differentiation, but also in the production of proinflammatory cytokines in fully differentiated adipocytes from human adipose-derived stem cells (hASCs), which may associate with AAP-induced weight gain^16^. Sertie *et al*. reported that in cultured hASCs, clozapine and olanzapine increased peroxisome proliferator-activated receptor gamma (PPAR-γ) expression and induced insulin-stimulated lipogenesis more than the typical antipsychotic drug haloperidol^17^.

Recent studies suggested that the sterol-regulatory element-binding proteins (SREBPs) are central in the regulation of various lipid biosynthetic pathways^18^. SREBPs are transcription factors that respond to nutrient levels and regulate the transcription of genes required for many aspects of lipid metabolism^19^, including genes that are important for downstream cholesterol and fatty acid biosynthesis^20^. There are three major SREBP isoforms, SREBP-1a, SREBP-1c (encoded by SREBF1 gene) and SREBP-2 (encoded by SREBF2 gene). SREBP-1 primarily controls the gene expression involved in fatty acid and triacylglycerol metabolism, whereas SREBP-2 preferentially regulates cholesterol metabolism^18^. SREBPs are synthesized as precursor proteins, which reside in the endoplasmic reticulum (ER), where they form a complex with SREBP chaperone proteins, an SREBP cleavage activating protein (SCAP) and an insulin-induced gene (Insig) protein^21^. At low sterol levels, SREBPs are released from the membrane and activated by cleavage; then, the water-soluble N-terminal domain is translocated to the nucleus^21, 22^ where it binds to the SRE in the promoter of numerous SREBP target genes for lipid biosynthesis^22^. Insigs have two isoforms; Insig-1, a target of nuclear SREBPs, whose mRNA expression depends on nuclear SREBP levels, and Insig-2, whose amount is low but constant and is negatively regulated by insulin without being influenced by SREBPs^18^. However, Yabe *et al*. reported that Insig-2 can also bind the SCAP/SREBP complex and cause its retention in the ER in a sterol dependent fashion. When sterols are present, Insig-2 causes ER retention of the SCAP/SREBP complex^23^. Liou *et al*. demonstrated that three haplotypes of the Insig-2 gene significantly increased the risk of AAP-induced metabolic syndrome. They also found that the Insig-1 gene was not associated with metabolic syndrome; however, the gene interaction between Insig-1 and Insig-2 was implicated in AAP-induced metabolic adverse effects^24^. Additionally, Le *et al*. also found a significant association between SREBP-mediated activation of lipid biosynthesis with Insig-2 blockade and weight gain during AAP treatment in patients with schizophrenia^22^.

The biochemical pathways involving SREBP may be altered by a variety of AAPs. AAPs, such as clozapine, olanzapine, and risperidone can elicit significant upregulation of SREBP-1 and SREBP-2 and their downstream target genes leading to increased lipid and cholesterol synthesis^25–28^. Several studies demonstrated that AAPs activate the SREBP which might be crucial for AAP-mediated dyslipidemia, weight gain and cardiovascular diseases^22, 24, 29, 30^. The work by Ferno and colleagues showed the possible of SREBP pathway activation in antipsychotic-induced metabolic disorders^26^. They demonstrated that the antipsychotic drugs clozapine and haloperidol both activate the SREBP pathway in cultured human glioma cells^26^. In other studies, clozapine induced pronounced activation of SREBP, with subsequent transcriptional activation of downstream cholesterol and fatty acid biosynthesis, in cultured hepatocytes and adipocytes^31, 32^. Additionally, both clozapine and olanzapine have been shown to enhance differentiation of adipose precursor cells 3T3-L1 into mature adipocytes, possibly through activation of SREBP-1^33^. However, little is known about the effects of AAPs on SREBP signaling and lipid biosynthesis in the early events of adipogenic differentiation in ASCs. Different AAPs are associated with various degrees of metabolic adverse effects^34^. Therefore, this study aimed to compare the effects of clozapine, olanzapine and risperidone on Insig/SCAP/SREBP pathway and the downstream expression of lipid biosynthesis genes during adipogenic differentiation without dexamethasone and insulin supplementation in rat ASCs.

## Results

### Effects of AAP treatment on cell viability

**Figure 1:**
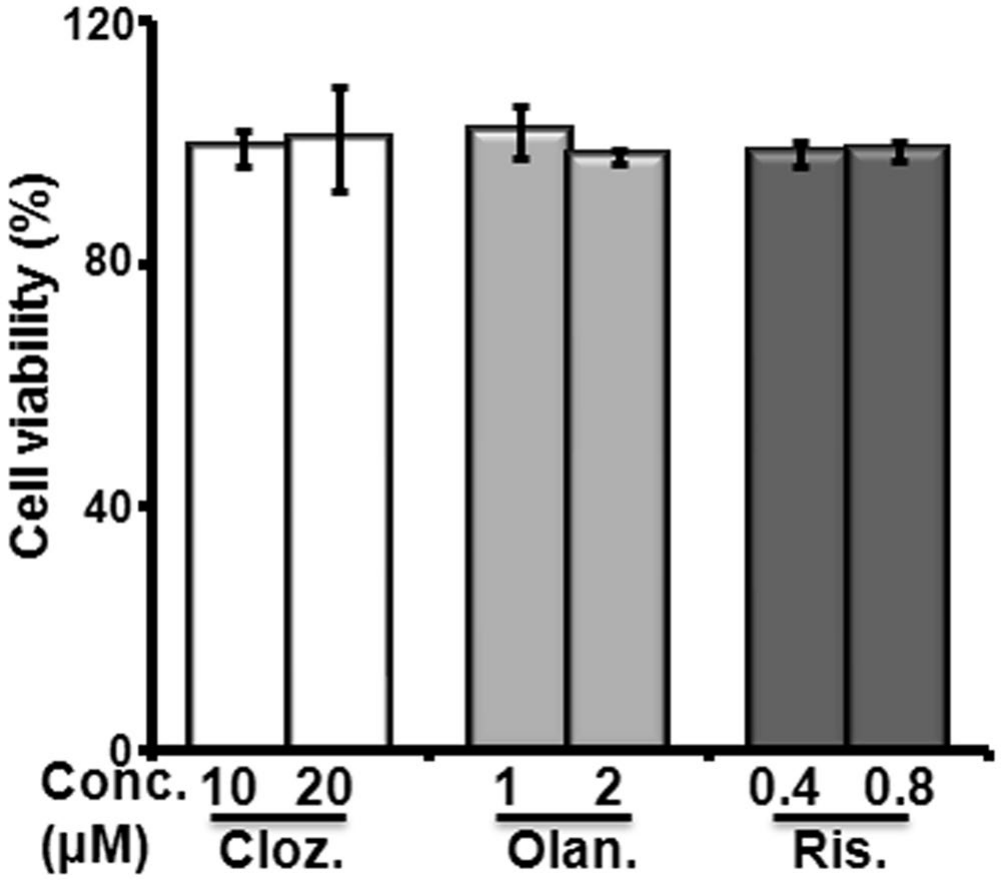
Effect of AAPs on cell viability. Cell viability was measured by CCK-8 assays. The cells were plated into 96-well microplates at a density of 5000 cells per well in complete medium and incubated at 37 °C in 5% CO_2_ prior to treatment. The cells were treated with clozapine (Cloz. 10 and 20 μM), olanzapine (Olan. 1 and 2 μM) and risperidone (Ris. 0.4 and 0.8 μM) for 48 hours. The data are representative of three independent experiments.

According to the therapeutic plasma concentrations of AAPs (0.6 to 1 μM for clozapine^35^, 0.07 to 0.2 μM for olanzapine^36^ and 10 to 40 nM for risperidone^37^), we have treated the cells with 10 to 20-fold higher concentrations of each AAP similar to a previous study^17^. To explore the effects of AAPs on cell viability, ASCs were cultured with clozapine, (10 and 20 μM), olanzapine (1 and 2 μM) or risperidone (0.4 and 0.8 μM) for 48 hours. AAPs at all concentrations tested did not affect the cell viability (Fig. 1). Moreover, there is no obvious difference of lipid biosynthesis genes by AAP treatment at day 7 (Supplementary Fig. 1). Therefore, clozapine at 10 μM, olanzapine at 1 μM and risperidone at 0.4 μM were applied in subsequent experiments.

### AAPs enhanced lipid droplet formation

**Figure 2:**
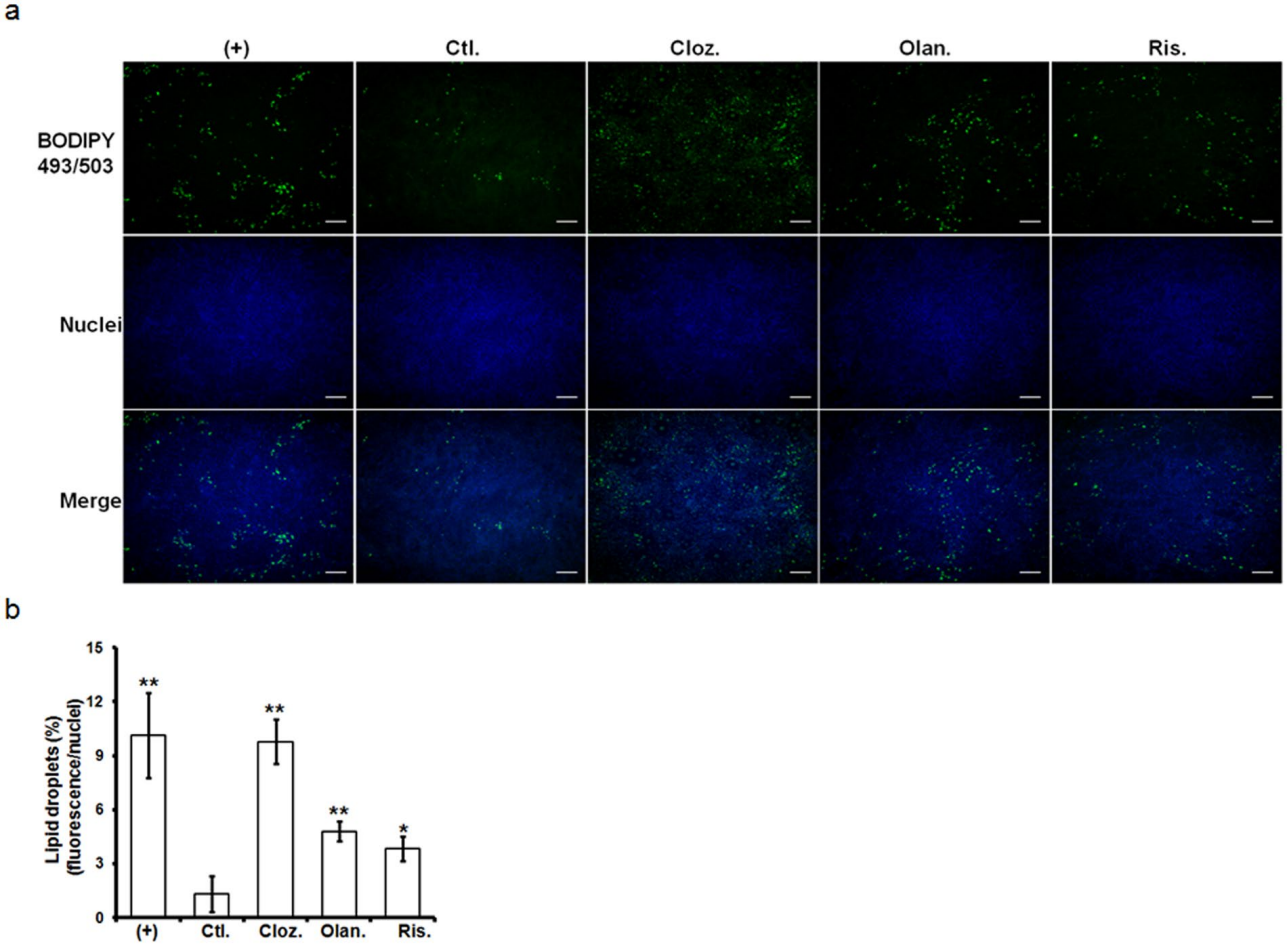
Effect of AAPs on lipid droplet accumulation in ASCs at day 7 after treatment. (**a**) Lipid droplets were stained with the BODIPY fluorescence probe. Cell nuclei were stained with DAPI and fluorescent images were taken at ×40. (**b**) Lipid vesicles were quantified via high-content imaging and normalized to cell number. (+): treated with adipogenic media (ADM) for the indicated times; Ctl.: treated with ADM for 2 days and changed to complete media only; Cloz.: clozapine; Olan.: olanzapine; Ris.: risperidone. Results are presented as the mean of four independent experiments ± SD. Scale bar: 20 μm. *, ** denotes statistical significance: *P* < 0.05 and 0.01, compared with the control group, respectively.

AAPs were tested for their effects on the adipogenic differentiation of ASCs. As shown in Fig. 2, clozapine, olanzapine and risperidone significantly enhanced the number of lipid vesicles compared with the vehicle-treated control.

### Differential modulatory effects of AAPs on SREBP signaling and lipid biosynthesis genes during adipogenesis

**Figure 3:**
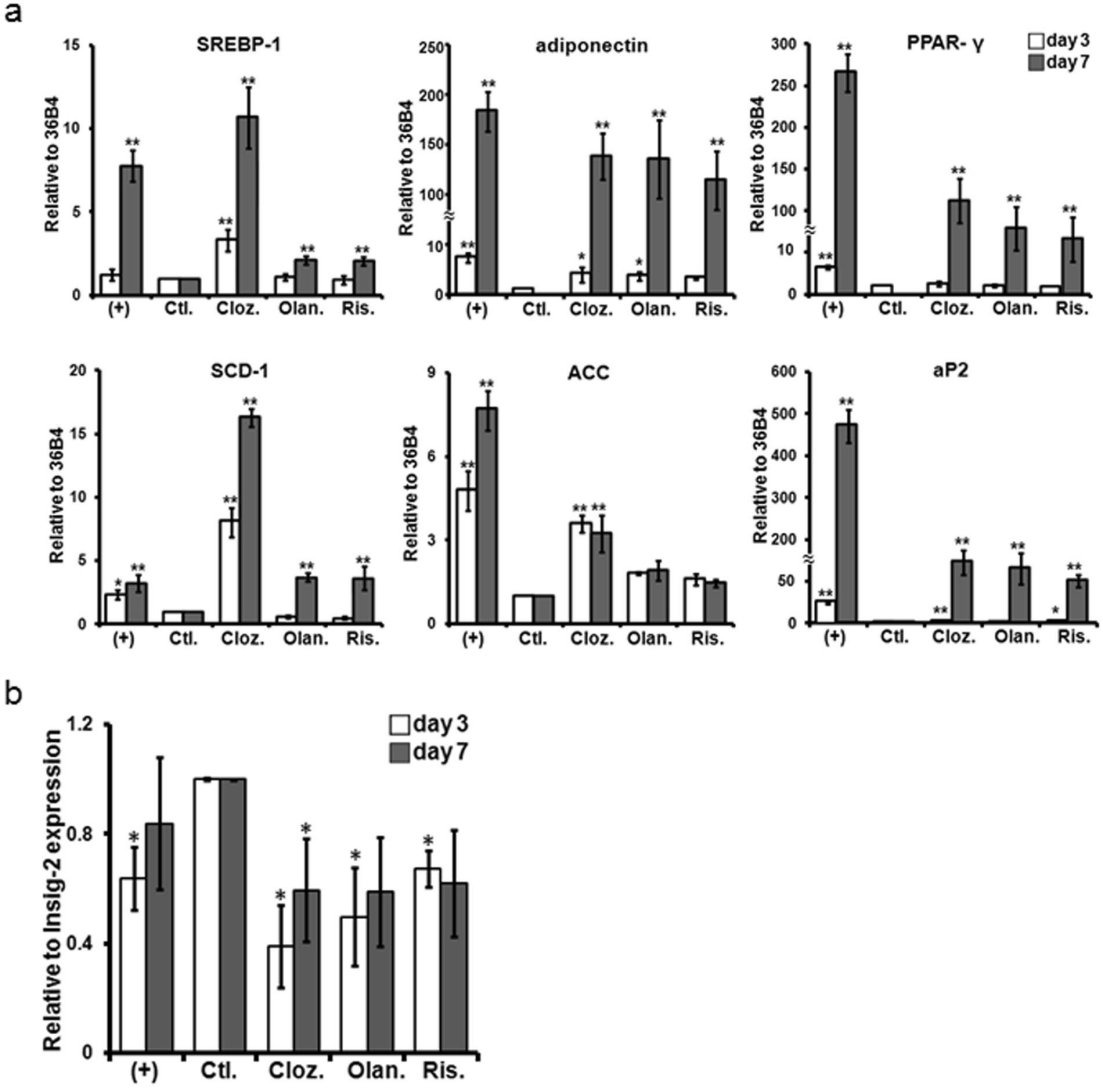
Effect of AAPs on lipid gene expression in ASCs at day 3 and day 7 after treatment. (**a**) SREBP-1 and its downstream lipid gene expression. (**b**) Insig-2 gene expression. ASCs were differentiated in the presence of AAPs (Cloz. 10 μM, Olan. 1 μM and Ris. 0.4 μM). At days 3 and 7, total RNA was extracted and subjected to q-PCR. The gene expressions levels were normalized to 36B4 expression. (+): treated with ADM for the indicated times; Ctl.: treated with ADM for 2 days and changed to complete media only; Cloz.: clozapine; Olan.: olanzapine; Ris.: risperidone. Results are presented as the mean of three independent experiments ± SD. *, ** denotes statistical significance: *P* < 0.05 and 0.01, compared with the control group, respectively.

We next examined the effects of AAPs on the control of the adipocyte differentiation program. Enzymes that are involved in free fatty acids (FFA) regulation, such as stearoyl-CoA desaturase (SCD-1), acetyl CoA carboxylase (ACC) and adipocyte fatty acid binding protein (aP2), were increased by clozapine treatment on day 3 of the differentiation process (Fig. 3a). Interestingly, clozapine treatment strongly enhanced SREBP-1 and SCD-1 expression compared with either the vehicle-treated control or positive control on days 3 and 7. On day 7, all AAP treatments increased the expression levels of SREBP-1, adiponectin, PPAR-γ, SCD-1 and aP2 compared with the vehicle-treated control (Fig. 3a). On the other hand, the ACC level was elevated by clozapine treatment only, suggesting that clozapine is an effective inducer of SREBP-mediated lipid synthesis under these experimental conditions. SREBP is known to regulate lipid biosynthesis when it leaves the ER membrane and enters the nucleus. Additionally, Insig-2 stabilizes SREBP on the ER membrane. As shown in Fig. 3b, Insig-2 gene expression was suppressed on day 3 in all AAP treatment groups as well as positive control, while it was also suppressed on day 7 by clozapine treatment only.

### AAPs reduced Insig-2 expression and enhanced SREBP activation

**Figure 4:**
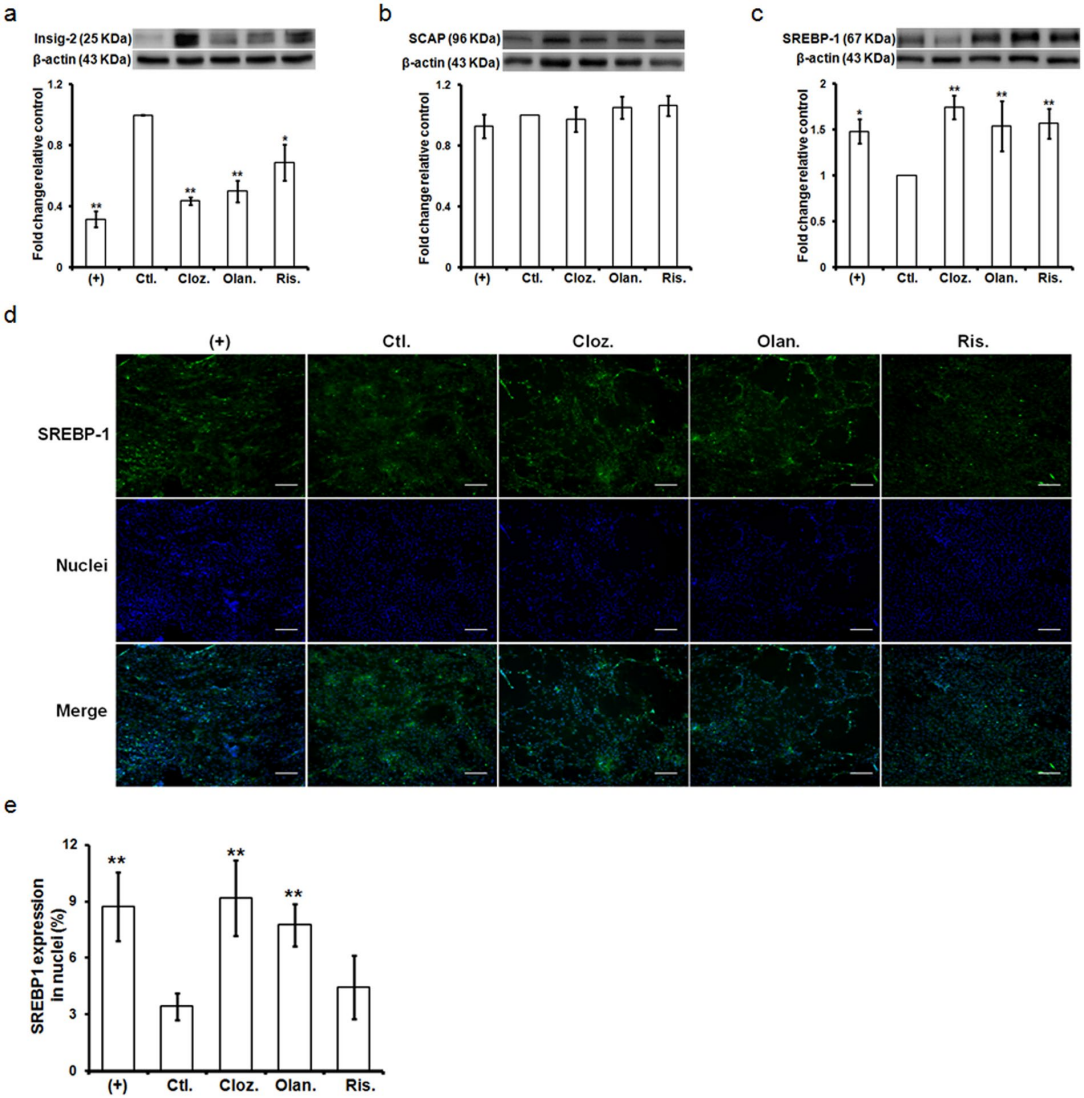
Effect of AAPs on SREBP signaling during adipogenesis. The cells were treated with ADM for 2 days and supplemented with AAPs for 7 days. (**a**–**c**) The cell lysates were subjected to SDS-PAGE and immunoprobed with antibodies against Insig-2, SCAP and SREBP-1. Each protein expression was normalized to the loading control (β-actin) and the data represent the expression ratio of each protein compared to the control group. Original scans are provided in Supplementary Fig. 3. (**d**) The nuclear SREBP-1 level was measured by immunofluorescence using an anti-SREBP-1 antibody. Cell nuclei were stained blue with DAPI and fluorescent images were taken at ×100. (**e**) Nuclear fluorescence intensity was quantified via high-content imaging. (+): treated with ADM for the indicated times; Ctl.: treated with ADM for 2 days and changed to complete media only; Cloz.: clozapine; Olan.: olanzapine; Ris.: risperidone. Results are presented as the mean of three independent experiments ± SD. Scale bar: 10 μm. *, ** denotes statistical significance: *P* < 0.05 and 0.01, compared with the control group, respectively.

SREBP is known to regulate lipid biosynthesis when it leaves the ER membrane and enters the nucleus. Additionally, Insig-2 stabilizes SREBP on the ER membrane. To verify whether AAPs regulate Insig/SCAP/SREBP signaling, we evaluated the protein levels of Insig-2, SCAP and SREBP-1 in cell lysates, as well as the nuclear level of SREBP-1, using immunoblotting and immunofluorescence microscopy. As shown in Fig. 4a–c, AAP treatment reduced Insig-2 expression but enhanced SREBP-1 expression compared with the vehicle-treated control. However, SCAP expression was not changed following AAPs treatment. The nuclear translocation of SREBP-1 was increased by clozapine and olanzapine treatment compared with the vehicle-treated control (Fig. 4d,e).

### Overexpression of Insig-2 suppressed adipogenic differentiation in ASCs

**Figure 5:**
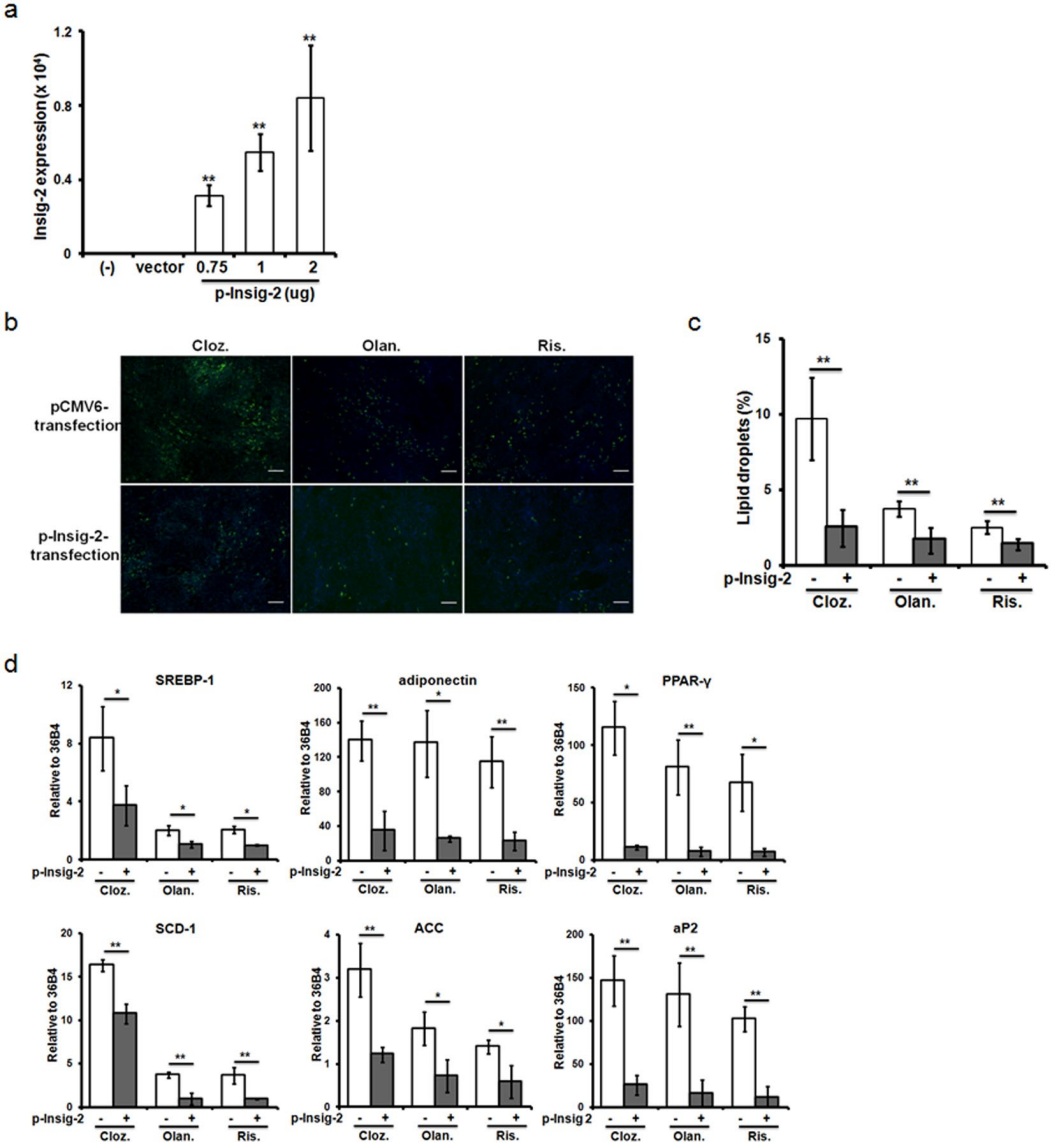
Overexpression of Insig-2 in ASCs during adipogenic differentiation. (**a**) The expression of Insig-2 in ASCs. ASCs were transfected with Insig-2 or the negative control pCMV6-Entry plasmid for 24 hours as described in the “Materials and Methods” section. (**b**) Lipid droplets were stained with the BODIPY fluorescence probe. Cell nuclei were stained blue with DAPI and fluorescent images were taken at ×40. (**c**) Lipid vesicles were quantified via high-content imaging. (**d**) The effects of AAPs on SREBP-1 and the downstream expression of lipid regulatory genes in Insig-2-transfected ASCs during adipogenic differentiation. The cells were switched to ADM for two days and then maintained in AAPs for 7 days. The gene expression was normalized to 36B4 and compared between the Insig-2-transfected group and the non-transfected group. Cloz.: clozapine; Olan.: olanzapine; Ris.: risperidone. Results are presented as the mean of three independent experiments ± SD. Scale bar: 20 μm. *, ** denotes statistical significance, *P* < 0.05 and 0.01 compared to the non-transfected group, respectively.

The Insig-2 gene contributes to the risk of metabolic syndrome independently and in an interactive manner with Insig-1 in schizophrenic patients treated with AAPs^24^. Additionally, AAP-treated rats showed significantly increased hepatic expression of SREBPs and corresponding inhibition of Insig-2 expression^25^. Next, we determined whether Insig-2 overexpression modulates lipid biosynthesis in ASCs. To evaluate the transfection efficiency of plasmid DNA in ASCs, the expression level of Insig-2 was validated by RT-PCR. The expression of Insig-2 was significantly up-regulated by plasmid DNA transfection for 24 hours compared with empty vector transfection (Fig. 5a). To determine whether increased expression of Insig-2 could affect the efficiency of adipogenic differentiation induced by AAPs in ASCs, we evaluated the lipid droplet formation and gene expression profiles. As shown in Fig. 5b,c, increased expression of Insig-2 significantly reduced the number of lipid vesicles compared to empty vector transfection following AAP treatment for 7 days. Moreover, increased expression of Insig-2 also inhibited the expression of lipid biosynthesis genes (Fig. 5d).

## Discussion

Although AAPs possess superior efficacy in treating both the positive and negative symptoms of schizophrenia compared to typical antipsychotics, AAP treatment promotes metabolic disorders and cardiovascular diseases^3–7^. Notably, both clozapine and olanzapine treatment lead to excessive weight gain with elevated fasting plasma triglyceride (TG) and cholesterol concentrations compared with other AAPs^38–40^. Additionally, Lauressergues *et al*. reported that clozapine directs its lipogenic effects specifically on the FFA and phospholipid (PL) pathway, whereas olanzapine causes more generally effects on FFA, PL, and TG synthesis in rat hepatocytes^27^. Yang *et al*. found that olanzapine increases TG accumulation and activates SREBP-1 during 3T3-L1 preadipocyte differentiation to the mature adipocyte phenotype^33^. In this study, we explored the effects of AAPs on adipogenic differentiation as well as its molecular consequences in ASCs. Our results showed that lipid droplet formation was strongly enhanced in response to clozapine treatment, but olanzapine and risperidone had moderate or less effect on day 7 after AAP treatment (Fig. 2). Moreover, clozapine treatment dramatically induced SREBP-1 and SCD-1 mRNA expression in the early phase of adipogenic differentiation (day 3) and adipose induction period (day 7). All AAPs increased SREBP-1 and its downstream lipid gene expression on day 7. The differential effects of AAPs on lipid droplet formation (clozapine > olanzapine > risperidone) may depend on the nuclear translocation of SREBP-1 induced by AAPs in adipogenesis (Fig. 4). Obesity is often associated with a low-grade state of inflammation, which is attributed to the production of inflammatory cytokines in adipose tissues and causes metabolic abnormalities^41^. AAP treatment activated nuclear factor kappa B (NF-κB) and caused a concerted increase in the levels of proinflammatory cytokines TNF-α, IL-1β and IL-8, and the MCP-1 in hASCs after adipose differentiation for 11 days. Their results showed that clozapine causes the greatest increase in the NF-κB, IL-1β and IL-8 expression levels^16^. Although AAP treatment activated NF-κB similar to the positive control for adipogenic differentiation (Supplementary Fig. 2), there is no obvious difference of proinflammatory cytokine expression in this study, probably due to short treatment period with AAPs for 3 and 7 days. Adipogenic differentiation induces dynamic changes in NF-κB expression and activity^42^. Therefore, NF-κB activation may be associated with adipogenic differentiation rather than proinflammatory response in this culture condition. In mammalian cells, PPAR-γ, and CCAAT/enhancer binding proteins (C/EBPs) are considered the key early regulators of adipogenesis^43^. PPAR-γ was known to be critical both for adipogenic differentiation and for maintenance of mature adipocytes^44, 45^. Sertie *et al*. has shown that clozapine and olanzapine enhanced the early (C/EBP-β and PPAR-γ) or late (PPAR-γ and LPL) markers of adipose tissue differentiation^17^. Further studies are necessary to clarify the impact of AAPs on early and late events of adipogenic differentiation as well as the effect of AAPs on adipocyte hypertrophy.

Several studies have shown a significant association between SREBP-mediated activation of lipid biosynthesis with Insig-2 blockade and AAP-induced weight gain in patients with schizophrenia^22, 24^. Additionally, many clinical studies have found that clozapine treatment had the highest risk for weight gain than other AAPs^38, 46^. The present study showed that clozapine significantly suppressed Insig-2 mRNA expression during adipogenic differentiation at early and induction phase (Fig. 3b). The results were consistent with the SREBP-1 and lipid gene expression after clozapine treatment (Fig. 3a). Additionally, AAP treatment significantly suppressed protein level of Insig-2 on day 7, which in turn enhanced SREBP-1 activity (Fig. 4). These results implicate Insig-2 in the pathogenesis of metabolic abnormalities in patients treated with AAPs. In support of our present data, Cervino *et al*. identified Insig-2 as a susceptibility gene for plasma cholesterol levels in mice^47^. In addition to the effect of Insig-2 on lipid metabolism, Herbert *et al*. demonstrated that a single-nucleotide polymorphism (SNP) of Insig-2 was associated with obesity^48^. A strong association between three Insig-2 gene SNPs (rs17587100, rs10490624 and rs17047764) and antipsychotic-related weight gain was reported^22^.

In this study, we demonstrated that overexpression of Insig-2 in ASCs suppresses SREBP-1 mRNA expression and subsequently inhibits AAP-induced lipid biosynthesis during adipogenic differentiation. In adult mice, deletion of the Insig genes activated SREBP-mediated lipogenesis in respiratory epithelial cells, resulting in lipotoxicity-related lung inflammation and tissue remodeling^49^. On the other hand, AAP treatment down-regulated Insigs and up-regulated SREBP expression in vitamin D-deficient rats. Vitamin D supplementation, in turn, increased Insig expression and abolished SREBP activation^50^. Interestingly, the active form vitamin D_3_ (1α,25(OH)_2_D_3_) directly suppresses adipogenic factors and inhibits adipocyte differentiation via strong induction of Insig-2 expression in 3T3-L1 cells^51^. Furthermore, 1α,25(OH)_2_D_3_, a novel response element in the promoter region of the Insig-2 gene, directs vitamin D receptor (VDR)-mediated transcriptional activation^51^. The above findings implicate the Insig proteins in the pathogenesis of AAP-induced adverse effects, including excessive weight gain and metabolic abnormalities; furthermore, these adverse effects were attenuated when the Insigs were modulated.

In summary, the present study demonstrates that AAP treatment significantly inhibits Insig-2 expression and, in turn, increases the expression of SREBP-1 and its downstream lipid genes during adipogenic differentiation in ASCs. Such abnormal lipogenesis was attenuated when Insig-2 expression was increased, suggesting that SREBP/Insig-2 signaling may be a therapeutic target for AAP-induced weight gain.

## Materials and Methods

### Drugs

AAPs including clozapine, olanzapine and risperidone, were purchased from Sigma-Aldrich (St. Louis, MO, USA). All drugs were dissolved in DMSO and stored at 4 °C until use.

### Ethics statement

All animal experimentation was in compliance with the animal welfare guidelines. All protocols were approved by Animal Protection Law by the Council of Agriculture, Executive Yuan, R.O.C., and the Guide for the Care and Use of Laboratory Animals by the Institute of Laboratory Animal Resources, National Research Council, USA.

### Preparation of rat ASCs

ASCs were obtained from the abdominal adipose tissue of 8-week-old Lewis rats as previously described^52^. Briefly, the tissue was minced, washed, and suspended in Hanks’ solution containing collagenase type II (Sigma-Aldrich). Following a 30–60 min digestion at 37 °C with agitation (50 rpm), which produced a smooth and even consistency, the cellular pellet was incubated with erythrocyte lysis buffer and resuspended in complete medium composed of DMEM supplemented with 10% fetal bovine serum (FBS, Thermo Fisher Scientific Inc. Carlsbad, CA, USA) and Penicillin-Streptomycin-Amphotericin B Solution (1%, Biological Industries, Cromwell, CT, USA). The cells were incubated for 3 to 6 days in a humidified 5% CO_2_ incubator until they reached approximately 80% confluence.

### Adipogenic differentiation assay

The ASCs were plated into 6-well plates and allowed to come to 60% confluence in growth media. After the cells were switched to adipogenic differentiation media (ADM) (DMEM supplemented with 10% FBS, 1 μM dexamethasone and 10 μg/ml insulin (Thermo Fisher Scientific Inc.)) for 2 days, the adipogenic media were removed and the cells were cultured in complete media supplemented with clozapine (10 and 20 μM), olanzapine (1 and 2 μM) or risperidone (0.4 and 0.8 μM) or in complete media without AAPs (as a vehicle-treated control) for 3 and 7 days. As positive and negative controls, the cells were cultured in ADM and in complete media, respectively.

### Cell viability assay

Cell viability was detected with the Cell Counting Kit-8 (CCK-8, Sigma-Aldrich), and the absorbance at 450 nm was measured using a Victor IV microplate reader (PerkinElmer, Waltham, MA, USA).

### Lipid droplet staining

After induction of adipogenic differentiation for 2 days and following AAP treatment for 7 days, cells were fixed in phosphate-buffered formaldehyde (4%; pH 7) for 15 min and stained with a BODIPY 493/503 fluorescence lipid probe for 15 min as previously described^53^. The nuclei were counterstained with 4’,6-Diamidino-2-Phenylindole (DAPI; Invitrogen, Thermo Fisher Scientific Inc.). Images of lipid droplets were captured using an inverted microscope (Olympus Corporation, Tokyo, Japan) or the ImageXpress^®^ Micro XLS Widefield High-Content Analysis System (Molecular Devices, Sunnyvale, CA, USA). The percentage of lipid droplets in each group was normalized to total number of cells determined by the DAPI counterstain. Data analysis was performed using the MetaXpress software package (Molecular Devices).

### Transformation of Insig-2 expression plasmids

Transformation of Insig-2-expressing pCMV-Insig-2 plasmids, obtained from OriGene (#RN211993, Rockville, MD, USA), was carried out in *Escherichia coli* DH5α, and DNA was prepared using plasmid purification columns (EndoFree Plasmid Giga Kit, Qiagen, Hilden, Germany). The plasmid DNA was dissolved in milli-Q water. The purified plasmid DNA was stored at −20 °C and diluted to 1 mg/mL with phosphate-buffered saline (pH 7.4) immediately before use.

### Transfection of the Insig-2 gene in ASCs

Transfection of the Insig-2 cDNA into ASCs was conducted when they reached 70% confluence. ASCs were transfected with Insig-2 or negative control pCMV6-Entry plasmids using the TurboFectin 8.0^TM^ High Performance Transfection Reagent (OriGene), following the manufacturer’s protocol. After 24 hours, ASCs were switched to ADM for two days, followed by AAP treatment for 7 days. ADM treatment was set as the positive control.

### RNA isolation and reverse transcriptase polymerase chain reaction (RT-PCR)

Total RNA was extracted using NucleoSpin-RNA kits (MACHEREY-NAGEL GmbH & Co. KG, Düren, Germany), and total RNA (3 μg) was reverse-transcribed into cDNA using the High-Capacity cDNA Reverse Transcription Kit (Applied Biosystems, Foster, CA, USA) according to the manufacturer’s instructions. The quantitative RT-PCR reaction was performed on an ABI 7500 Fast Real-Time PCR System with the SDS 1.4 program (ABI 7500 Fast PCR system; Applied Biosystems) using the SYBR Green PCR Master Mix. Reverse transcriptase (RT)-negative samples were used to demonstrate that the signals obtained were RT-dependent. The 36B4 reference gene was used to normalize the data. The 2^−∆CT^ value, which corresponds to the expression ratio of each gene compared to 36B4, and the 2^−∆∆CT^ value, which corresponds to the expression ratio of each gene compared to the vehicle-treated control group, were calculated. The sequences of gene-specific primers were summarized in Supplementary Table 1.

### Western blot analysis

The proteins were separated on 10% SDS-PAGE gels and transferred onto PVDF membranes (Bio-Rad). The membranes were blocked with 5% (w/v) skim milk/1% (v/v) Tween 20 in PBS for 30 min at room temperature and incubated overnight with the appropriate primary antibody: Insig 2 from Biorbyt (Biorbyt Llc, SF, CA, USA, 1:500 dilution); SCAP from Bioss (Bioss Inc., Woburn, MA, USA, 1:1000 dilution); SREBP1 antibody from BioVision (BioVision Inc., Milpitas, CA, USA, 1:500 dilution); NF-κB p65 (Santa Cruz Biotechnology, Santa Cruz, CA,USA, 1:1000 dilution); phospho-NF-κB p65 (Cell Signaling Technology, Inc., Danvers, MA, USA, 1:1000 dilution); anti-β-actin antibody from Millipore (Millipore Corporation, Bedford, MA, USA, 1:10000 dilution) at 4 °C. Detection was performed using an enhanced chemiluminescence (ECL) detection kit (Millipore). Images were captured using a G:BOX Image Station iChemi XL device (SYNGENE, Cambridge, UK), and the relevant bands were quantified by densitometry using GeneTools (SYNGENE).

### Immunofluorescence staining

Intracellular SREBP-1 staining was performed using Image-iT® Fix Perm Kit (Thermo Fisher Scientific Inc.). Briefly, cells were fixed and permeabilized in Fixation Buffer and Permeabilization Buffer for 15 min, respectively. The cells were blocked and incubated with a primary monoclonal antibody against SREBP-1 (BioVision Inc., 1:100 dilution). After incubation with fluorophore-conjugated secondary antibodies (Thermo Fisher Scientific Inc.), cells were counterstained with DAPI (Invitrogen, Thermo Fisher Scientific Inc). After washing with PBS, cells were examined using an FV10i confocal laser microscope (Olympus) or the ImageXpress^®^ Micro XLS Widefield High-Content Analysis System (Molecular Devices). Data analysis was performed using the MetaXpress software package (Molecular Devices).

### Statistical analysis

Descriptive statistics, means, standard deviations, and ranges were used where appropriate. For comparison of groups, one-way analysis of variance (ANOVA) and Duncan’s post-hoc test were used where appropriate. Differences between the non-transfected and transfected groups were tested for statistical significance using Student’s *t*-test. The results are shown as the mean value ± the standard deviation (SD) of the mean. A value of *p* < 0.05 was considered significant.

## Electronic supplementary material

Supplementary Information
