# Supplementary material for: Overexpression of Insig-2 inhibits atypical antipsychotic-induced adipogenic differentiation and lipid biosynthesis in adipose-derived stem cells
Source: Sci Rep. 2017 Sep 7;7:10901. doi: 10.1038/s41598-017-11323-9 (PMC5589828; doi:10.1038/s41598-017-11323-9)
Supplement: Supplementary file 1 — Supplementary Information [file 41598_2017_11323_MOESM1_ESM.pdf]

SUPPLEMENTARY INFORMATIONS:

Overexpression of Insig-2 inhibits atypical antipsychotic-induced adipogenic differentiation and lipid biosynthesis in adipose-derived stem cells

Chien-Chih Chen[1,3], Li-Wen Hsu[2], Kuang-Tzu Huang[2], Shigeru Goto[2], Chao-Long Chen[2] and Toshiaki Nakano[2,3*]

[1]Department of Psychiatry, [2]Liver Transplantation Center and Department of Surgery, Division of Transplant Immunology, Institute for Translational Research in Biomedicine, Kaohsiung Chang Gung Memorial Hospital and Chang Gung University College of Medicine, Kaohsiung, 833, Taiwan

[3]Graduate Institute of Clinical Medical Sciences, Chang Gung University College of Medicine, Kaohsiung, 833, Taiwan

* To whom correspondence may be addressed. E-mail: nakano33@mail.cgu.edu.tw

TEL: +886-7-731-7123 (Ext.8190); FAX: +886-7-733-6856

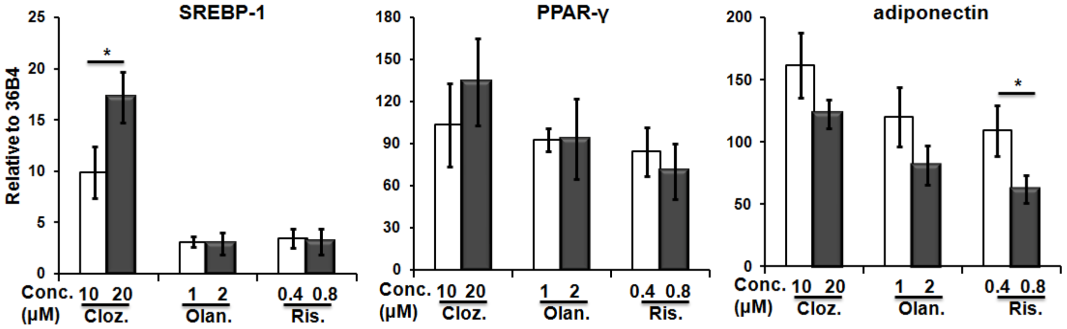

Supplementary Figure 1. Concentration responses of AAPs on lipogenic gene expressions. ASCs were differentiated in the presence of AAPs (Cloz. 10 and 20 μM; Olan. 1 and 2 μM; Ris. 0.4 and 0.8 μM). At days 7, total RNA was extracted and subjected to q-PCR. The gene expressions levels were normalized to 36B4 expression and compared to the vehicle-treated control group, respectively.

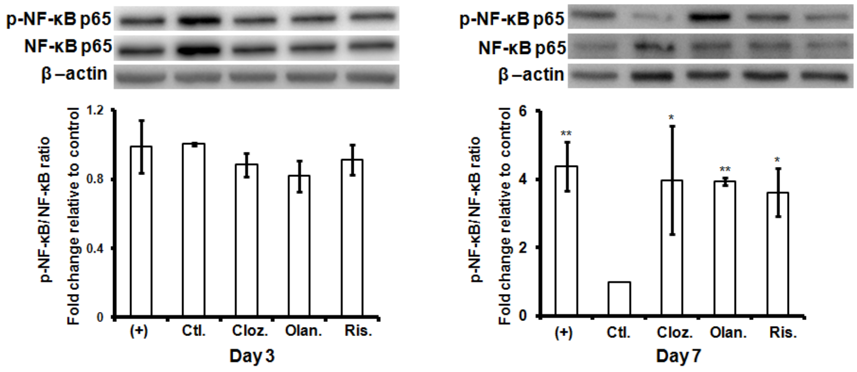

Supplementary Figure 2. Effect of AAPs on NF-κB activation during adipogenesis. The cells were treated with ADM for 2 days and supplemented with AAPs for 3 and 7 days. The cell lysates were subjected to SDS-PAGE and immunoprobed with antibodies against phospho-NF-κB and NF-κB. Each protein expression was normalized to the loading control (β-actin). Proteins expression were probed on same blot. (+): treated with ADM for the indicated times; Ctl.: treated with ADM for 2 days and changed to complete media only; Cloz.: clozapine; Olan.: olanzapine; Ris.: risperidone. Results are presented as the mean of three independent experiments ± SD.

(a)

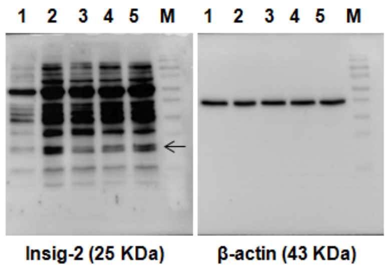

(b)

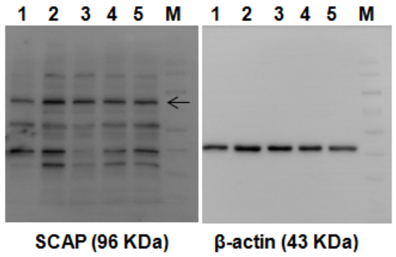

(c)

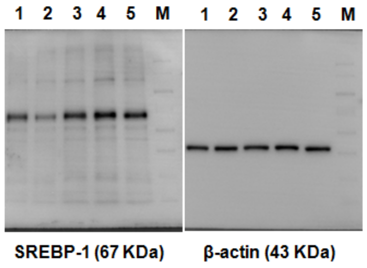

Supplementary Figure 3. Effect of AAPs on SREBP signaling during adipogenesis. The cells were treated with ADM for 2 days and supplemented with AAPs for 7 days. (a-c) The cell lysates were subjected to SDS-PAGE and immunoprobed with antibodies against Insig-2, SCAP and SREBP-1. Lane 1: (+): treated with ADM for the indicated times; Lane 2: Ctl.: treated with ADM for 2 days and changed to complete media only; Lane 3: clozapine; Lane 4: olanzapine; Lane 5: risperidone; M: marker.

Table S1. qRT-PCR primer sequences.

Gene name	Genebank accession no.	Locations of primers (5'-3')	Fragment size (bp)
adiponectin	NM_144744	Forward: GATACCGGGCCGTGATGG<br>Reverse: CCCTTCCGCTCCTGTCATTC	118
ACC	NM_022193	Forward: TCCCGGAGCTACTCTTAAAAAATG<br>Reverse: CCCCAACGCCCACATG	71
aP2	RNU75581	Forward: TCCTTCAAACTGGGTGTGGAA<br>Reverse: CCAGGGTTATGATGCTCTTCACT	73
SREBP1	XM_213329	Forward: CATCGACTACATCCGCTTCTTACA<br>Reverse: GTCTTTCAGTGATTTGCTTTTGTGA	96
SCD-1	NM_139132	Forward: CAACACCATGGCGTTCCA<br>Reverse: GCGTGTGTCTCAGAGAACTTGTG	81
Insig 2	NM_178091	Forward: CGCTCTTGGTTGCCATGTA<br>Reverse: TGATGAGATTTTTGAGCAATAACTTT	107
36B4	NM_022402	Forward: TTCCCACTGGCTGAAAAGGT<br>Reverse: CGCAGCCGCAAATGC-3'	60
GAPDH	NM_017008	Forward: TTCTAGAGACAGCCGCATCT<br>Reverse: TGGTAACCAGGCGTCCGATA	106
